# The Effect of Foam Rolling for Three Consecutive Days on Muscular Efficiency and Range of Motion

**DOI:** 10.1186/s40798-018-0141-4

**Published:** 2018-06-08

**Authors:** Lewis J. Macgregor, Malcolm M. Fairweather, Ryan M. Bennett, Angus M. Hunter

**Affiliations:** 1 Faculty of Health Sciences and Sport, University of Stirling, Stirling, UK; 2 SportScotland: Scottish Institute of Sport, Stirling, UK

**Keywords:** Electromyography, Maximal voluntary contraction, Self-massage, Tensiomyography

## Abstract

**Background:**

Foam rolling (FR) has been shown to alleviate some symptoms of exercise-induced muscle damage and has been suggested to increase range of motion (ROM) without negatively impacting strength. However, it is unclear what neuromuscular effects, if any, mediate these changes.

**Methods:**

In a randomized, crossover design, 16 healthy active males completed 2 min of rest or FR of the knee extensors on three consecutive days. Mechanical properties of vastus lateralis (VL) and rectus femoris (RF) were assessed via Tensiomyography. Knee extension maximal voluntary contraction (MVC) and knee flexion ROM were also assessed, and surface electromyography amplitude (RMS) was recorded during a submaximal isometric contraction (50% of MVC). Measures were performed before and after (0, 15, and 30 min) FR or rest.

**Results:**

MVC was reduced on subsequent days in the rest condition compared to FR (*p* = 0.002, _p_*η*^2^ = 0.04); ROM was not different across time or condition (*p* = 0.193, _p_*η*^2^ = 0.01). Stiffness characteristics of the VL were different on the third day of FR (*p* = 0.002, _p_*η*^2^ = 0.03). RMS was statistically reduced 0, 15, and 30 min after FR compared to rest (*p* = 0.006, _p_*η*^2^ = 0.03; *p* = 0.003, _p_*η*^2^ = 0.04; *p* = 0.002, _p_*η*^2^ = 0.04).

**Conclusions:**

Following FR, MVC was elevated compared to rest and RMS was transiently reduced during a submaximal task. Excitation efficiency of the involved muscles may have been enhanced by FR, which protected against the decline in MVC which was observed with rest.

## Key Points

- FR reduced the muscle excitation required to complete a submaximal task, which led to reduced fatigue after 3 days. Muscle mechanical properties were altered following 3 days of FR.
- FR may be used for a sustained period to alter muscle mechanical properties and to enhance muscular efficiency.
- Participants were all healthy and active, so it is not clear if FR would have the same effect among clinical populations.

## Background

In recent years, self-massage through foam rolling (FR) has become an increasingly popular treatment in managing muscular injuries and pain [1–3]. FR involves an individual applying their own body weight to a neoprene-coated cylinder, using small repetitive undulating movements to exert pressure on the muscle. Potential mechanisms of action for FR can be divided into two categories: first mechanical, focussed around alterations in the structure or state of fascial tissue; or second neurophysiological, focussing on afferent signaling from mechanoreceptors [4]. As the majority of FR research has focused on functional outcomes, or has involved FR subsequent to exercise-induced muscle damage, it remains difficult to ascertain which, if any, of these proposed mechanisms holds true.

Specific aims of FR have emerged around enhancing recovery and sporting performance [5, 6]. Observed effects of FR have included reduced delayed-onset muscle soreness following physical activity [7, 8]. It has also been reported that there are reduced decrements in muscle function subsequent to a bout of eccentric exercise, when treated with FR [9]. Also, FR has reduced knee extensor electromyography (EMG) amplitude during a dynamic lunge, which may be interpreted as improved movement efficiency due to lower excitation being required for a given task [10]. However, to date, there has been little research into the application of FR as a preventative as opposed to a recovery tool [9, 11]. Moreover, there remains much conjecture around the overall efficacy of FR, with mixed findings regarding the acute effect on muscle function [12, 13] and range of motion (ROM) [14–16].

Evidence suggests that combining deep tissue massage with static stretching results in reduced spinal reflex excitability without altering twitch contractile properties during treatment, while stretching alone can prolong electromechanical delay, which remains unaffected by massage [17]. Therefore, whilst stretching increases ROM through both neural and mechanical factors, massage-induced alterations can more likely be attributed to reflex inhibition [17]. However, in our laboratories, we have linked massage to muscle force impairment alongside maintained neuromuscular recruitment [18]; as a result of this, we proposed that observed force impairment was due to changes in muscle architecture and associated alterations in series compliance. While these architectural changes were not measured in the aforementioned study [18], such alterations would likely affect muscle mechanical properties [19], which can be measured from the extent of muscle radial displacement, through tensiomyography (TMG) [20]. To date, only two studies [21, 22] have measured muscle mechanical properties, using TMG, on rested muscle, following FR, both studies reporting no acute change in muscle displacement, with measurements performed following a single session of FR. Given that anecdotal evidence suggests FR is typically used by athletes habitually performing prolonged and high-intensity exercise to break up trigger point adhesions and alleviate perceived muscle stiffness, it is important to asses if FR can attenuate exercise-induced changes in contractile properties, following repeated applications. However, to our knowledge, no study has examined the effect of more prolonged application of FR following exercise, across consecutive days, on muscle contractile properties.

If FR is to be effectively prescribed to treat muscle injuries and pain [5, 23–25], it is necessary to understand the mechanisms of action, in order to establish best practice. Therefore, the aim of the present study was to investigate muscle mechanical properties, strength and ROM over the course of three consecutive days of treatment with well-controlled bouts of FR. It was anticipated that the repeated demands of the testing procedure should induce detectable residual fatigue, as may be expected with day-to-day submaximal exercise. However, increased neuromuscular efficiency through FR would limit the symptoms of fatigue. Therefore, we hypothesized that FR would reduce muscle stiffness characteristics and increase ROM, without additional force decrement costs, in spite of fatigue-associated impairments in muscle function.

## Methods

### Participants

Based on a priori power analysis (G*Power 3) of predicted changes in ROM following self-massage, with *α* = 0.05 and 1-β = 0.80, sixteen healthy, recreationally active, male participants with no history of neuromuscular or musculoskeletal disorders were recruited to complete this study (age 24.5 ± 4.4 years, height 1.8 ± 0.1 m, body mass 82.4 ± 8.0 kg, and knee extension strength (MVC) at baseline 222.0 ± 36.5 N m). Participants were not currently undertaking any form of self-massage at the time of their participation in the study and were provided with no prior indication as to the hypothesis regarding the potential effects of self-massage. Participants refrained from (1) any unaccustomed physical activity for the duration of the trial and (2) any strenuous exertion for at least 24 h prior to each testing session. Volunteers provided written consent, having been informed of any potential risks involved in their participation. The study was performed in accordance with the standards set by the latest revision of the Declaration of Helsinki and was approved by the local Research Ethics Committee.

### Study Design

**Fig. 1:**
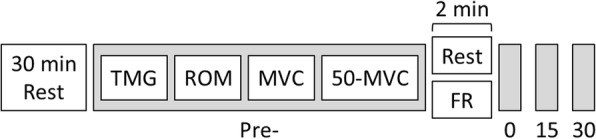
Timeline of the experimental design. TMG = tensiomyography; ROM = range of motion; MVC = maximal voluntary contraction; 50-MVC = 50% of maximal voluntary contraction; FR = foam rolling

Following full familiarization of the testing procedures (utilizing the non-dominant leg), participants reported to the laboratory for two separate trials in a randomized, counterbalanced crossover design to incorporate two different interventions; one intervention was foam rolling while the other was in the rested condition. Participants were assigned, via block randomization, to decide the order in which trials were completed. Each trial consisted of three testing sessions on three consecutive days, with 7 days separating the start of each trial. Participants reported to the laboratory following an overnight fast and initially rested in a supine position for 30 min. Following this rest period, mechanical and contractile properties of the vastus lateralis (VL) and rectus femoris (RF) were measured using tensiomyography (TMG: BMC Ltd., Ljubljana, Slovenia). Participants were then tested for knee flexion range of motion (ROM) before isometric strength assessments, using an isokinetic dynamometer (Kin Com, Chattanooga, Hixson, TN, USA). Following baseline measures, participants either rested for 2 min or performed 2 min of self-massage (FR). All measurements were then repeated, in an identical order to pre-intervention: immediately, 15-min and 30-min post rest/FR (Fig. 1). All measurements were performed on the dominant leg. Dietary intake records were completed on the day preceding each session of the first trial, and participants were instructed to replicate their dietary intake before each visit for the second trial.

### Protocol

**Fig. 2:**
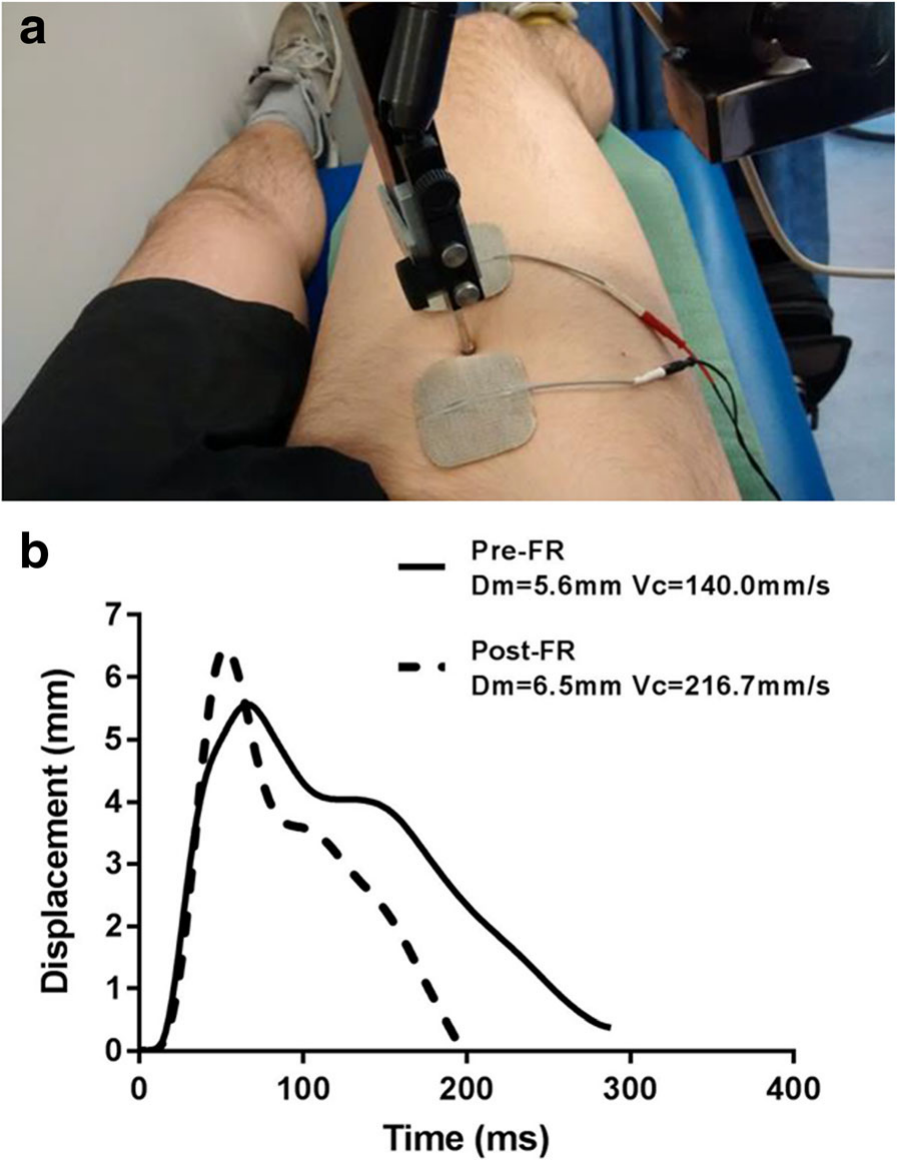
**a** Positioning of electrodes and displacement sensor for tensiomyography measurement (rectus femoris). **b** Typical displacement/time curve of the tensiomyographic signal pre- and 30-min post-foam rolling. FR = foam rolling; Dm = muscle displacement; Vc = contraction velocity

After resting in a supine position for 30-min participants then adopted a knee joint angle of 60° (0^o^ = full extension), which was maintained by the use of a foam support placed beneath the popliteal fossa. Two pairs of self-adhesive electrodes (5 cm^2^) (Axelgaard, USA) were affixed to the skin: one pair over the VL and one pair over the RF (Fig. 2a). A digital TMG displacement sensor (GK 40, Panoptik d.o.o., Ljubljana, Slovenia) was then positioned perpendicular to the muscle belly, equidistant between the pair of electrodes [26]. The sites over each muscle, of the sensor and the electrode pair, were marked with semi-permanent ink to enable exact relocation following FR treatment and on subsequent days [27, 28]. A single 1-ms wide stimulation pulse was delivered, which applied initial current amplitude of 20 mA. This amplitude was progressively increased by 10 mA increments until peak twitch response was obtained [26]. In order to minimize the effects of fatigue and potentiation, rest periods of 10 s were allowed between each stimulation pulse. Typical peak responses were observed at amplitude between 40 and 70 mA, and only the output data for that particular stimulation intensity were used for analysis. Output parameters were extracted and analyzed from each peak twitch response [27]: Displacement (Dm), the extent of maximal radial deformation (mm) of the muscle belly during contraction; Contraction velocity (Vc), the rate (mm/s) of contraction between 10 and 90% of maximal displacement [Vc = Dm80/Tc] where Tc = contraction time between 10 and 90% of peak radial displacement of the muscle belly; Dm80 = the radial displacement occurring during the time period of Tc [26] (Fig. 2b).

Knee flexion ROM was measured in accordance with previous literature [9]. Participants adopted a modified kneeling lunge position. The non-dominant leg was positioned with the sole of the foot flat on the floor and the knee flexed to 90°; participants were permitted to place their hands on this knee for support, but were instructed to angle their torso perpendicular to the floor throughout the ROM assessment. With the hip of the dominant leg extended as far as possible, the foam roller was placed under the ankle in order to standardize the starting position. Internal knee angle was recorded, using a goniometer, and then, the knee was flexed as far as voluntarily possible. Maximal knee flexion was held only as long as was required to measure the internal angle for a second time. Total ROM was taken as the difference between starting knee angle and end knee angle. Intraclass correlation coefficient (ICC) between baseline ROM at the start of each trial was 0.85.

Participants were coupled to the isokinetic dynamometer and secured using nylon straps, according to the manufacturer’s guidelines. Gravitational corrections were performed, in accordance with existing recommendations [29]. A pair of Ag/AgCl self-adhesive electrodes (PNS Dual Element Electrode; Vermed, VT, USA) were affixed to the skin over the VL, ^1^/_3_ of the distance from the greater trochanter to the lateral femoral epicondyle, with an inter-electrode distance of 20 mm, following thorough preparation of the skin in accordance with SENIAM guidelines [30]. A reference electrode was affixed to the patella. These electrodes remained in place throughout the entire duration of the testing session. Surface EMG (sEMG) was recorded during all isometric contractions and was synchronized with torque output. sEMG was captured at 2 KHz, anti-aliased with an upper bandwidth of 500 Hz and band pass filtered automatically at 10–500 Hz using a fourth-order zero-lag Butterworth filter.

Participants performed a standardized warm up [31, 32] prior to baseline measurements. Maximal isometric voluntary contraction (MVC) was measured at a knee joint angle of 60° (0° = full extension); the limb was secured by a Velcro strap proximal to the medial malleolus. The angle of 60° was chosen, as it lies within the well-established range of reported optimal knee joint angles, for peak isometric torque production [33]. Participants were instructed to exert peak force as quickly as possible and to hold each contraction for 5 s. Consistent verbal encouragement was provided by the same investigator throughout, to ensure maximum effort. To maintain internal validity, the same two investigators were present throughout all testing [34]. Participants performed three contractions with 60-s recovery between each. The highest force output achieved was designated MVC and stored for analysis. ICC between baseline MVC at the start of each trial was 0.70. Participants next performed a 30-s submaximal isometric contraction at 50% of their baseline MVC (50-MVC). Torque was to be increased gradually until the target output was reached then held as steady as possible for 30 s (mean torque ± SD: 104.2 ± 10.7 N m and 102.7 ± 10.1 N m, during rest and FR trials respectively). Participants were provided with visual and verbal feedback throughout 50-MVC. This protocol was expected to result in moderate, but detectable symptoms of residual muscle fatigue, across the duration of the testing period. sEMG was captured for 30 s during 50-MVC; signals were RMS converted using the data collection software (Acknowledge® 3.9.1, Biopac Systems Inc., Goleta, CA, USA) and normalized to sEMG RMS from the MVC. Normalized RMS were divided into 5 × 6 s epochs for analysis.

### Foam Rolling

**Fig. 3:**
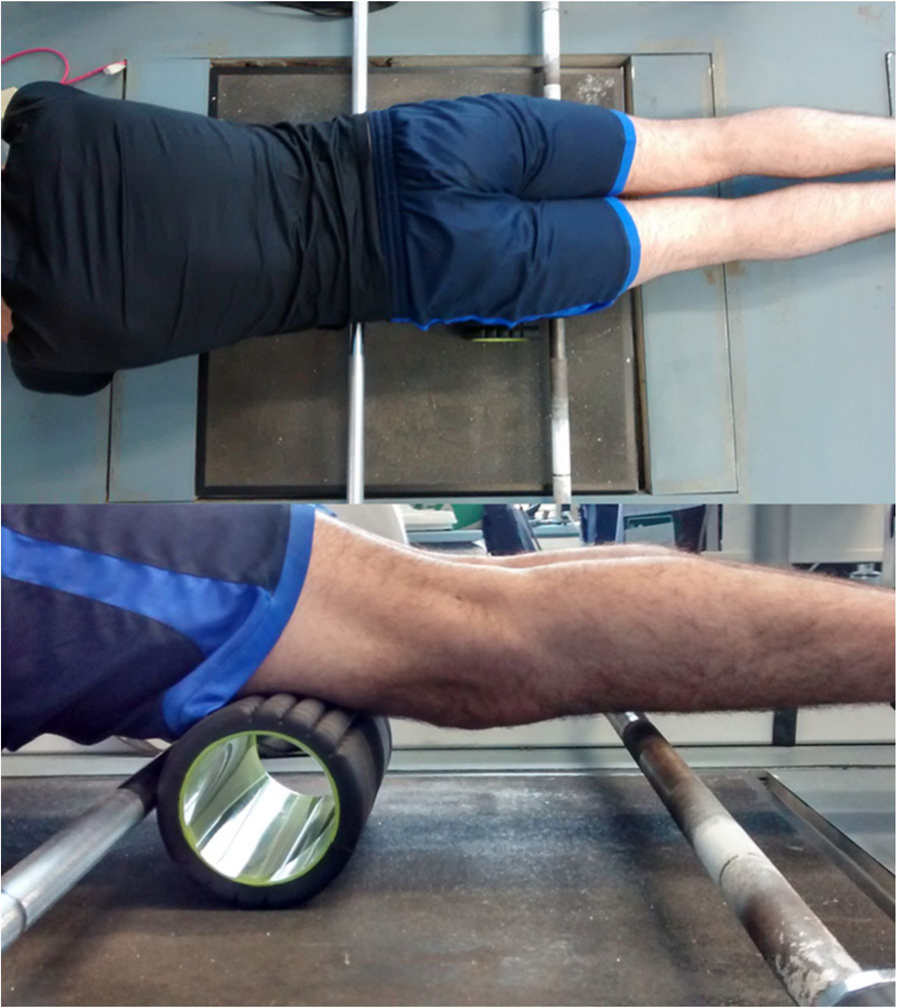
A participant performing self-massage using the foam roller

Following baseline measurements, participants performed self-massage, using a commercially available foam roller (TriggerPoint Performance, Austin, TX, USA) constructed of a hollow PVC pipe surrounded by a thin layer of neoprene [35]. The foam roller was positioned initially at the mid-point between the anterior inferior iliac spine (AIIS) and the upper border of the patella; participants supported themselves upon their forearms. To ensure that there were no other ground contact points, participants were instructed to plantar flex, whilst also positioning their feet and knees together, in order to focus the pressure of the foam roller upon the anterolateral aspect of the thigh (Fig. 3). The length of area that was treated with FR was ^2^/_3_ of the distance between the AIIS and the upper border of the patella. A custom-built metal frame positioned beneath the participant ensured that the correct area was treated. Once in position, participants rolled backwards and forwards in an undulating motion; the rate of movement was controlled by a metronome set at a predetermined rate based on one complete roll of the treated area (proximal-to-distal or distal-to-proximal) per 1 s. The foam roller was exclusively in contact with a force platform (400S Force Plate, Innervations, Australia), and force was recorded throughout the 2-min FR treatment using Acqknowledge® software at a sampling frequency of 2 KHz (Table 1). It has been suggested that stretching- and massage-based interventions may elicit a whole-body systemic response, such that treating one limb may nullify the effectiveness of the contralateral limb as a comparison [21, 24, 36]. Therefore, a separate (rest) condition was completed; however, as the timeframe of any residual effect of FR has not been established, the start of the rest condition and FR trials were seperated by 7 days. During the rest condition, participants adopted a supine position with the popliteal fossa of their dominant leg resting upon the foam roller in order to maintain a knee joint angle of ~ 60° (0° = full extension). The duration of the rest period was identical to the duration of FR.

**Table 1:** Force applied during FR treatment

	D0	D24	D48	Overall
Average force rolled (N)	48.2 ± 54.4 (1.13)	45.3 ± 50.6 (1.12)	48.0 ± 81.6 (1.70)	49.3 ± 62.9 (1.28)
Average relative force rolled (N/Kg)	7.0 ± 1.3 (0.19)	6.6 ± 1.0 (0.15)	6.0 ± 0.9 (0.15)	6.5 ± 1.1 (0.17)

*FR* foam rolling

Values are mean ± SD (Coefficient of Variation %), *n* = 16

### Statistical Analysis

All data were tested for assumption of homoscedasticity (Levene’s test) and normality (Ryan-Joiner test); residuals were assessed for linearity and normal distribution. Comparisons were performed between normalized RMS values (as described above); all other comparisons were performed using un-normalized data, analyzed using a three factor repeated measures (group [2] × time [4] × day [3]) analysis of variance (ANOVA) for normalized data; when analyzing un-normalized data, baseline values were included as covariates (ANCOVA). Tukey post hoc analysis was performed where appropriate (Minitab 16 statistical software, Minitab Ltd., Coventry, UK). Statistical significance was accepted at *p* < 0.05. All values were reported as mean ± standard deviation (SD), and partial *η*^2^ effect sizes (_p_*η*^2^) were calculated by _p_*η*^2^ = SS_conditions_/(SS_conditions_ + SS_error_).

## Results

### Maximal Isometric Voluntary Contraction and Range of Motion

**Fig. 4:**
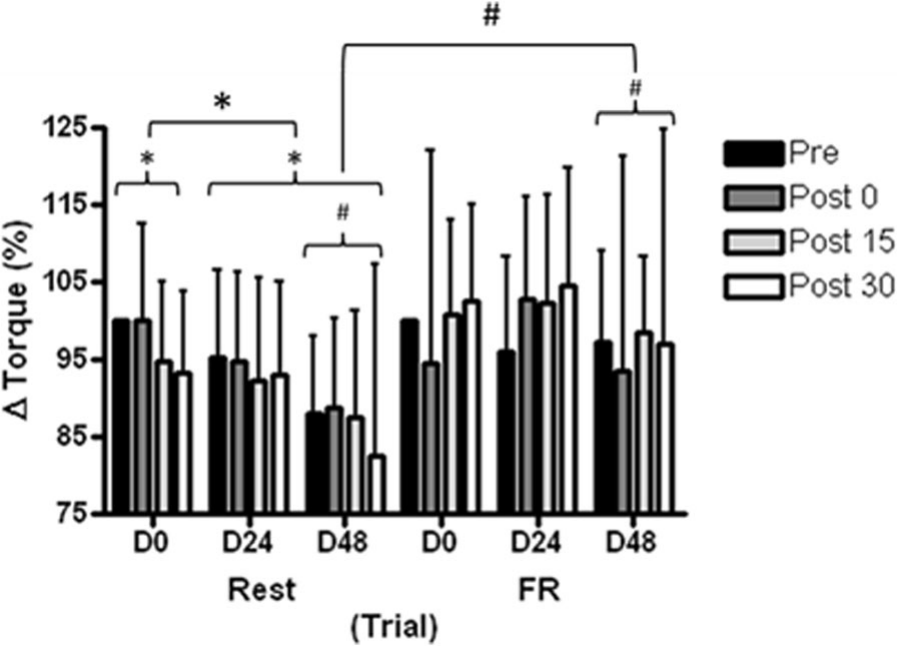
Maximal isometric voluntary contraction (MVC) of the knee extensors in the control (Rest) and intervention (FR) conditions. Values are mean ± SD, *n* = 16. *Statistically significant within condition differences, *p* < 0.001. #Statistically significant difference between conditions, *p* < 0.001. FR = foam rolling

A statistically significant interaction effect (*F*_(2,15)_ = 6.53, *p* = 0.002, _p_*η*^2^ = 0.04) was detected in MVC across days and condition (Fig. 4); this was caused by the rest condition statistically declining over the full-time period whereas the FR was maintained (*p* = 0.024). ROM was not statistically different between conditions or over time (*F*_(3,15)_ = 1.58, *p* = 0.193, _p_*η*^2^ = 0.01).

### Neuromuscular Recruitment

**Fig. 5:**
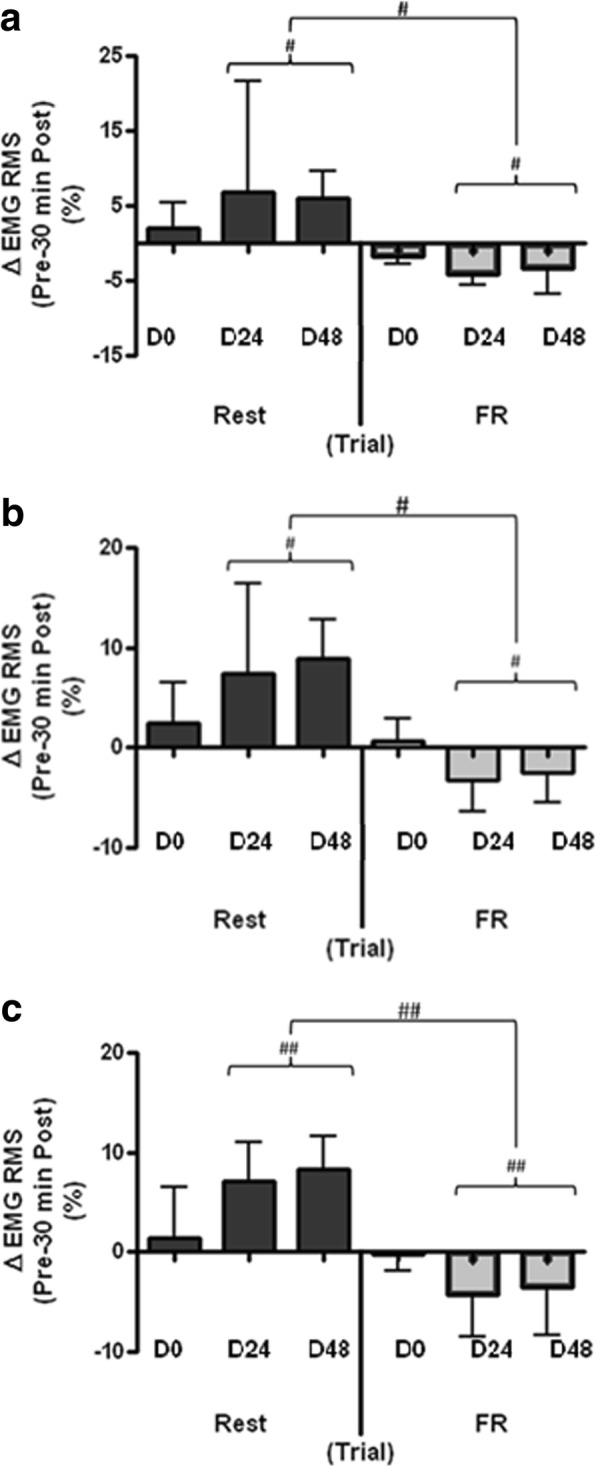
Change in normalized RMS (**a** 0–6, **b** 12–18, and **c** 24–30 s) from pre- to 30-min post-treatment in the control (Rest) and intervention (FR) conditions. Values are mean ± SD, *n* = 16. #Statistically significant difference between conditions, *p* < 0.01; ##Statistically significant difference between conditions, *p* < 0.001. FR = foam rolling; EMG RMS = electromyography root mean squared

On D24 and D48, FR statistically reduced RMS required for 30-s submaximal contraction, compared to rest. This reduction was observed during 0–6 s (*F*_(2,15)_ = 12.62, *p* < 0.001, _p_*η*^2^ = 0.07), 12–18 s (*F*_(2,15)_ = 20.68, *p* < 0.001, _p_*η*^2^ = 0.1) and 24–30 s (*F*_(2,15)_ = 24.37, *p* < 0.001, _p_*η*^2^ = 0.1), with statistically significant interaction effects (*F*_(3,15)_ = 4.18, *p* = 0.006, _p_η^2^ = 0.03; *F*_(3,15)_ = 4.71, *p* = 0.003, _p_*η*^2^ = 0.04; *F*_(3,15)_ = 5.21, *p* = 0.002, _p_*η*^2^ = 0.04) respectively between time and condition. These were caused by statistically reduced RMS immediately, 15- and 30-min post-FR compared to post-rest (*p* = 0.031; *p* = 0.049; *p* = 0.039). Figure 5 shows the change in RMS between pre and 30 min post on each trial day in both rest and FR.

### Contractile Properties

**Fig. 6:**
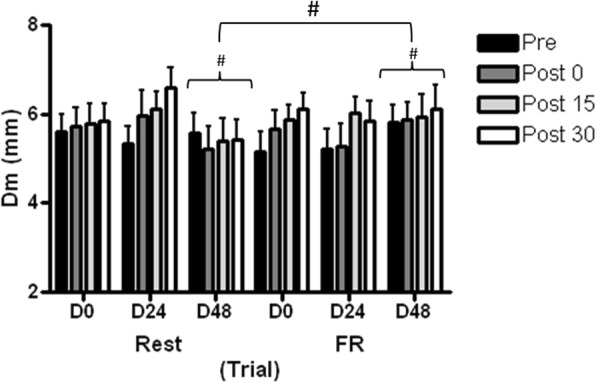
Maximal radial displacement (Dm) of vastus lateralis (VL) muscle belly. Values are mean ± SD, *n* = 16. #Statistically significant difference between conditions, *p* < 0.001. FR = foam rolling; Dm = muscle displacement

A statistically significant interaction effect was detected in peak displacement (Dm) (*F*_(2,15)_ = 6.32, *p* = 0.002, _p_*η*^2^ = 0.03) and velocity (Vc) (*F*_(2,15)_ = 4.87, *p* = 0.008, _p_*η*^2^ = 0.02) of VL with day and condition (Fig. 6), caused by statistically greater Dm (*p* = 0.021) and Vc (*p* = 0.023) on D48 in the FR treatment condition compared to the rest condition. Dm (*F*_(2,15)_ = 2.17, *p* = 0.116, _p_η^2^ = 0.01) and Vc (*F*_(2,15)_ = 1.61, *p* = 0.202, _p_*η*^2^ = 0.009) of RF were not statistically different between conditions or over time.

## Discussion

This study demonstrated reduced EMG RMS, during sustained submaximal contraction, following FR, when compared to increased RMS following rest. MVC was maintained following FR, avoiding decreases observed throughout the rest condition. Contractile characteristics of RF were unaffected, as shown by unaltered TMG parameters, although reduced muscle stiffness characteristics, and increased contraction velocity were evident in VL following the third consecutive day of FR. It had been hypothesized that elevated Dm would be indicative of altered muscle architecture and thus be associated with augmented ROM; surprisingly however, ROM remained unchanged over time and did not differ between conditions.

Perhaps the greatest insight into the mechanisms behind the effects of FR has been provided by investigating neuromuscular recruitment during static fatiguing contractions; RMS was lower at all time points following FR compared to the same time points following rest. Within the rest condition, RMS increased from baseline up to 15 and 30 min post-rest, potentially indicating that the muscle required greater neural drive in order to maintain 50% of MVC [37], suggesting that submaximal fatigue occurred [38]. However, similar changes in RMS arise due to peripheral factors, such as metabolite accumulation [39]. Increased neural drive is indicated by increased RMS which occurs as higher threshold motor units (MU) are recruited to sustain force production, in compensation for earlier recruited MUs becoming fatigued [40, 41]. In agreement with our hypothesis, these findings suggest that FR was able to reduce the impact of fatigue during this submaximal task, allowing the muscle to be activated more efficiently. Furthermore, on D48, RMS of the rest condition was higher than the previous 2 days, in addition to being elevated vs. FR condition, indicating there may have been chronic adaptation to repeated bouts of FR on consecutive days. However, given the constraints discussed above, it cannot be confirmed that changes in RMS were the result of altered neural drive, as opposed to peripheral factors. Future research should therefore incorporate M-wave amplitude characteristics, and normalize EMG to M-wave, in order to neutralize the effect of peripheral muscle fatigue, and isolate factors relating to neuromuscular efficiency [39, 42, 43].

It seems plausible that sustained muscle lengthening under pressure, applied during FR, will stimulate muscle spindles and Golgi tendon organ [10, 17]. Stimulation of these mechanoreceptors would lead to increased activity of type Ib afferents, thereby leading to greater proprioceptive feedback from the muscle in question to the CNS, as has been demonstrated through altering muscle-spindle length following proprioceptive neuromuscular facilitation (PNF) [44, 45]. The viscoelastic nature of skeletal muscle also may impact responses to FR [46, 47]. Indeed, shear wave velocity of knee flexors has been shown to decrease following FR; while this was not associated with increased ROM, the combination of FR with active warm up prolonged observed increases in ROM beyond active warm up alone [48].

Peak torque (MVC) was elevated 30 min after FR, which was also higher than 30 min post-rest. The decline in MVC on D24 and D48 of the rest condition can be interpreted as another marker of the fatiguing effect of the test protocol. It follows that lower neural drive required to complete the submaximal task following FR, compared to rest, indicates less demand on the muscle and, as such, has enabled maintenance of MVC following consecutive days of FR. Previous studies have reported no alteration in strength or performance [6, 49] or improved performance through combining FR with a dynamic warm up compared to a dynamic warm up alone [13]. The present study is the first to report an improvement in MVC following FR alone. Previous studies have measured strength or performance ≤ 10 min after FR; in accordance, we found no change in MVC immediately and 15 min post-FR. When force output is important, these findings suggest that for optimal effect, FR should be performed ≥ 30 min prior to activity.

As FR has previously been reported to increase ROM without decrements in force production, one of the main aims of this study was to examine the contractile mechanics of muscles subjected to FR. It was hypothesized that muscle displacement would increase following consecutive days of FR, indicative of reduced muscle stiffness. Despite the lack of improvement in ROM, higher displacement was observed in VL on D48 of FR treatment compared to D48 of the rest condition. Furthermore, Dm was 15.7% greater than baseline, 15 min post-FR, and 19.0% greater than baseline 30 min post-FR on D48; compared to 3.7% lower and 2.8% lower at 15 and 30 min post-rest. Although no differences were revealed in RF, there was a tendency towards greater displacement following FR. Similarly, Murray et al. [21] reported no change in ROM; however, they also reported no effect of FR on TMG Dm, although only a single bout of FR was performed. Given that we observed a difference in Dm between groups only on D48, it may be speculated that continuing with FR treatment for a more extended period of time may lead to more pronounced effects on contractile properties. It should also be noted however, that in the present study, independent of day, VL displacement was greater 15 and 30 min post treatment, in both the rest and FR conditions. Therefore, it seems that the test protocol itself may have impacted upon muscle mechanical properties; in order to fully elucidate the impact of FR on mechanical properties, investigation of the impact of FR on muscle contractile properties in isolation from other measurements may be required, to avoid interference from additional stretch and fatigue. Furthermore, other researchers have described mixed results following FR protocols lasting between 1 and 3 weeks [50, 51]; a dose response may exist, such that prolonged FR treatment, lasting ≥ 1 week, may prove more effective than acute FR, or treatments lasting ≤ 6 days. Interestingly, shorter treatment periods, combining FR with complimentary techniques, such as static stretching [51] or active warm up [48] may augment the response. As such, future research should aim to explore the optimal combination of techniques to elicit neuromuscular outcomes.

## Conclusions

This is the first study to illustrate elevated strength 30 min after a 2-min bout of FR, alongside reduced RMS during submaximal activity following FR. This reduced RMS protected the muscles from the fatiguing effects of the protocol, observed through the rest condition. Muscle displacement was increased after three consecutive days of FR; however, this did not translate into improved ROM. It seems that a single bout of FR may lead to alterations in neural drive. Such alterations potentially enhance strength and performance and delay the onset of fatigue.

## Abbreviations

Dm: Displacement
FR: Foam rolling
MVC: Maximal voluntary contraction
_p_η^2^: Partial eta squared
RF: Rectus femoris
RMS: Root mean squared
ROM: Range of motion
sEMG: Surface electromyography
TMG: Tensiomyography
Vc: Contraction velocity
VL: Vastus lateralis
